# P-1898. Reduction of Blood Culture Contamination Rates and Associated Healthcare Costs Through Implementation of Certified Phlebotomist-Drawn Blood Cultures at a Community Teaching Hospital

**DOI:** 10.1093/ofid/ofae631.2059

**Published:** 2025-01-29

**Authors:** Shikha Mishra, Michael Valdez, Danna Mejia, Adonica Vickers, Royce H Johnson, Carlos M D’Assumpcao, Rasha Kuran, Michelle Fang

**Affiliations:** Kern Medical-UCLA, Bakersfield, California; Kern Medical, Bakersfield, California; Kern Medical, Bakersfield, California; Kern Medical, Bakersfield, California; Kern Medical Center, Bakersfield, California; Kern Medical, Bakersfield, CA, Bakersfield, California; Kern Medical Center, Bakersfield, California; Kern Medical, Bakersfield, California

## Abstract

**Background:**

Blood culture contamination (BCC) is a drain on healthcare resources, with clinical implications such as delayed diagnosis. At Kern Medical (KM), a safety net teaching hospital in Bakersfield, CA, BCC in some areas had continually exceeded the 3% recommended maximum, with 59% of new positive blood cultures per patient representing contamination in early 2023. In response, a quality improvement initiative was launched in 2023 to reduce BCC through certified phlebotomist-drawn blood cultures (PDBC).

**Methods:**

A retrospective cohort study was conducted to assess the clinical and financial impact of BCC reduction through PDBC. Any blood culture with growth of a common commensal organism during the first 2 months of 2024 was reviewed for attributable hospital length of stay (LOS) and ED, laboratory, procedure and pharmacy resources associated with BCC. Patients were excluded if blood culture growth was indicative of true infection or if the patient was still admitted at the time of review or transferred to another hospital. Costs were calculated based on literature and institution-specific charges.

**Results:**

BCC was identified in 35 patients over the study period, with coagulase-negative *Staphylococcus* (CoNS) species isolated in 71% of patients and polymicrobial growth in 34% of cases. Forty percent of patients had been admitted with a non-infectious principal diagnosis, and 14% of patients had been discharged at the time of blood culture positivity and were readmitted or called back to the ED for management of possible bacteremia. Transthoracic echocardiogram had been performed for assessment of infective endocarditis in 34% of all patients and 40% of patients with CoNS prior to final identification of the blood culture isolate(s), which required 5.5 days on average. Sixty-six percent of patients received vancomycin, including 43% of patients admitted for noninfectious diagnoses. Twenty-three percent of patients had new or additional LOS due to BCC, with an average of 1.4 days. The average additional cost associated with a BCC event at KM was $2931.

**Conclusion:**

Given that BCC rates decreased at KM from 4.6% with non-certified phlebotomy staff collection compared to 1.2% with certified phlebotomist collection, introduction of PDBC resulted in an estimated cost avoidance of $778,596 per year.

**Disclosures:**

All Authors: No reported disclosures

